# Intraoperative optical coherence tomography in descemet stripping automated endothelial keratoplasty: pilot experiences

**DOI:** 10.1007/s10792-016-0338-9

**Published:** 2016-09-21

**Authors:** Jasper G. Steverink, Robert P. L. Wisse

**Affiliations:** Department of Ophthalmology, University Medical Center, Heidelberglaan 100, 3508GA Utrecht, The Netherlands

**Keywords:** DSAEK, Intraoperative optical coherence tomography, Live OCT, Posterior lamellar keratoplasty, RESCAN

## Abstract

**Purpose:**

To assess the added value of intraoperative optical coherence tomography (iOCT) in evaluating graft adhesion and graft interface in patients undergoing descemet’s stripping automated endothelial keratoplasty (DSAEK).

**Methods:**

This is a prospective single-center case series comprising 8 eyes of 8 patients consecutively scheduled for DSAEK surgery. iOCT imaging was performed after insertion of the graft, after pressurizing the eye, and at the end of surgery (three images per surgery). At each stage of surgery, corneal thickness and the widest gap between the recipient and the graft (i.e., maximal interface width) were measured using an image processing tool. Follow-up measurements were taken at 1 day, 3 and 6 months, post-operatively.

**Results:**

Imaging was performed in 21 of 24 scheduled imaging intervals, and required little to no additional surgical time. At the end of surgery, iOCT showed persisting interfaces in six cases. One case showed a full graft detachment necessitating surgical intervention.

**Conclusion:**

Real-time iOCT is a safe, efficient, and useful tool in assessing graft adherence in DSAEK surgery. With adequate analysis software, iOCT has the potential to be a paradigm-shifting development in posterior lamellar surgery and could aid the clinician in further lowering the rates of graft dislocation after DSAEK.

## Introduction

Posterior lamellar keratoplasty has replaced penetrating keratoplasty (PKP) as standard intervention for Fuchs’ endothelial dystrophy and bullous keratopathy [1, 2]. Of these, posterior lamellar keratoplasties, descemet stripping (automated) endothelial keratoplasty (DS(A)EK) [3] is one of the most widely used techniques [1, 4]. A more recent development is the addition of intraoperative optical coherence tomography (iOCT) in endothelial keratoplasties. iOCT enables the surgeon to see beneath the surface of the cornea in a cross-sectional plane with micrometer resolution. While the use of OCT in posterior eye surgery is well documented, iOCT in the anterior eye is still an upcoming field. Knecht et al. reported on iOCT in DSAEK surgery using an external hand-held device [5], and literature on the use of a fully integrated iOCT system (Rescan 700, Carl Zeiss AG, Oberkochen, Germany) in DMEK is emerging [6–8]. Post-operative OCT to assess graft thickness and OCT-aided intraoperative measuring of interface width have been reported, using several iOCT systems including Rescan 700 [9–12]. This study describes the results of a pilot study on the additional value of the Rescan 700 during DSAEK surgery in assessing graft adhesion by tracing persisting interface fluid and graft thickness at various time points during and after the surgery.

## Materials and methods

### Study design

A prospective single-center case series was performed at the University Medical Center, Utrecht, the Netherlands. All consecutive cases scheduled for DSAEK surgery for endothelial failure between March 12 and March 19 were included in this study.

The study was approved by the institutional ethical review board and complied with local laws and the principles of the Declaration of Helsinki. Pre- and post-operative measurements included uncorrected and best corrected visual acuity (UCVA/BCVA), manifest refraction, intra-ocular pressure (IOP), slit-lamp examination, central pachymetry (Pentacam HR; Oculus GmbH; Wetzlar, Germany), endothelial cell density counts (ECD; SP-3000, Topcon, Tokyo, Japan), and anterior segment imaging (Visante OCT; Carl Zeiss AG, Oberkochen, Germany).

### Intraoperative OCT device

A commercially available platform was used, where iOCT is fully integrated in the ophthalmic surgical microscope (Rescan 700, Carl Zeiss AG, Oberkochen, Germany). The platform is based on the Lumera 700 microscope, and the live OCT images can be projected in a *heads up* fashion in one of the oculars. The OCT engine used is a Spectral Domain-OCT producing 27.000 A-scans per second. The light source used is a superluminescent diode (SLD) with a central wavelength of 840 nm (bandwidth = 90 nm). The scan depth is 2.0 mm in tissue with an axial resolution of 5.5 µm and transversal resolution of 15 µm [13]. The Rescan 700 is one of currently two commercially available integrated systems, together with the Haag-Streit system (Haag-Streit Surgical, Wedel, Germany).

### Graft lenticule measurements

**Fig. 1:**
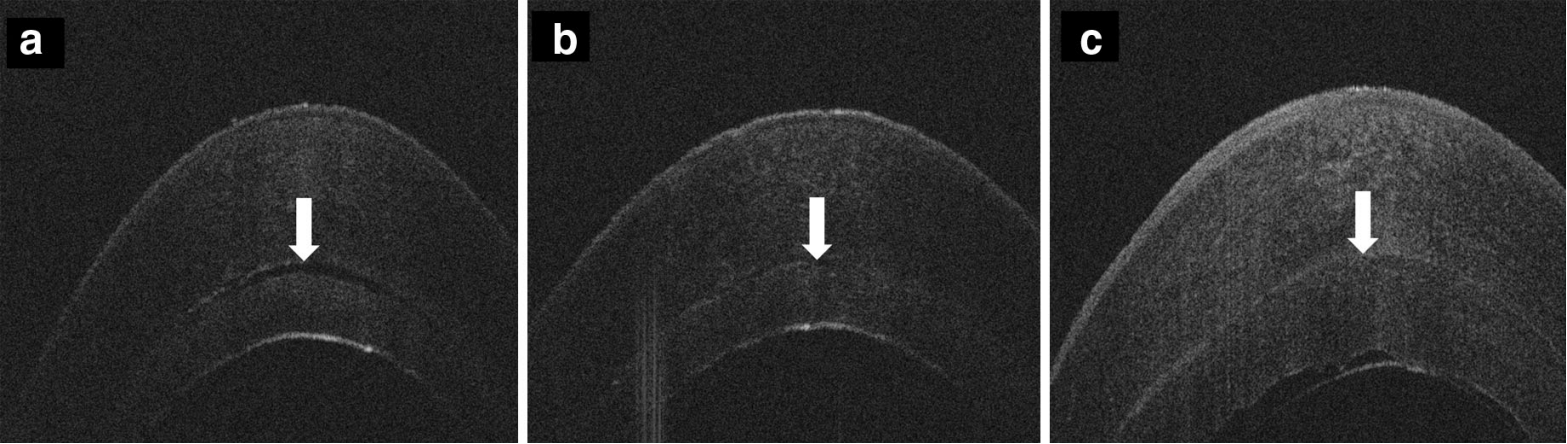
**a** Lamella and recipient cornea directly after insertion of the graft. The *arrow* indicates the interface. **b** Pressurizing the eye with an air bubble. The interface width decreases. **c** After 12 min of pressure when the IOP is normalized. The graft is fully adhered and no persisting interface is seen. An air bubble, the size of the graft, is left in place

Initial graft lenticule thickness measurements were supplied by the Euro Cornea Bank (Beverwijk, the Netherlands) as measured with anterior segment OCT (Casia SS-100, Tomey, Nagoya, Japan). The live video stream and OCT images were recorded with special details of three moments in surgery: directly after the insertion of the lamella (Fig. 1a), after pressurizing the eye to adhere to the lamella (Fig. 1b), and after 12 min of maximum pressure when the IOP is normalized (Fig. 1c). Post-operative graft thickness was measured with the anterior segment OCT at 1 day, 3 and 6 months.

### Surgical technique

DSAEK surgery was performed as described by Melles et al. [3] with precut corneal grafts provided by the Cornea bank. The graft size used was 8.5 mm in all cases. The graft was injected into the anterior chamber using either a Tan Endoglide inserter (AngioTech, Reading, PA/Network Medical Products, North Yorkshire, UK) or a reusable Macaluso inserter (Janach Instruments, Como, Italy). Adherence to the recipient stroma was maintained by an air bubble and IOP of ca. 65 mmHg. After 12 min, the IOP was normalized and the air bubble, the size of the graft, was left in place. If a considerable fluid in the interface was detected with iOCT, corneal swiping was performed as described by Price et al. [14]. Venting incisions were not made. The patient remained supine for 4 h after surgery.

### Analysis

**Graph 1:**
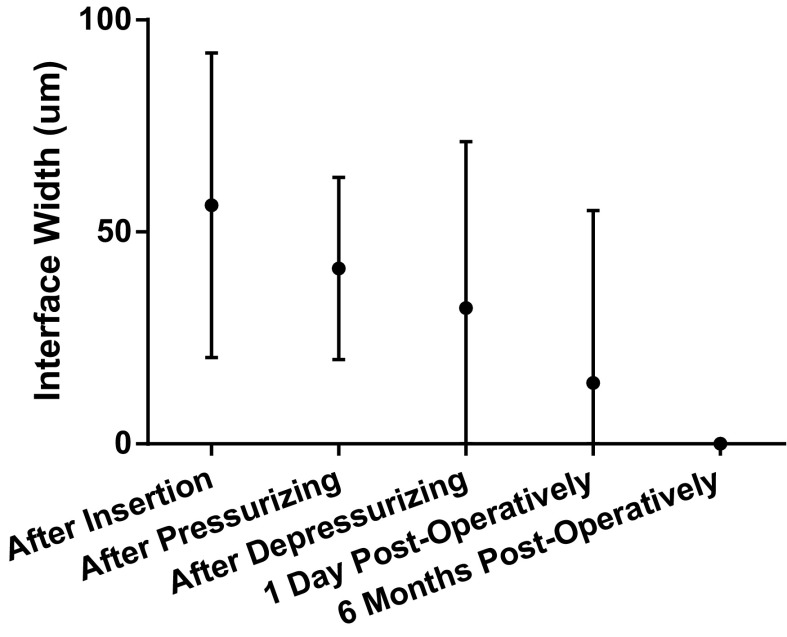
Column graph of mean interface widths (in µm) with 95 % confidence intervals. Interface width was measured directly after insertion of the graft, directly after pressurizing the eye, after 12 min of pressure when the IOP is normalized, on day 1 post-operatively, and 6 months post-operatively

**Graph 2:**
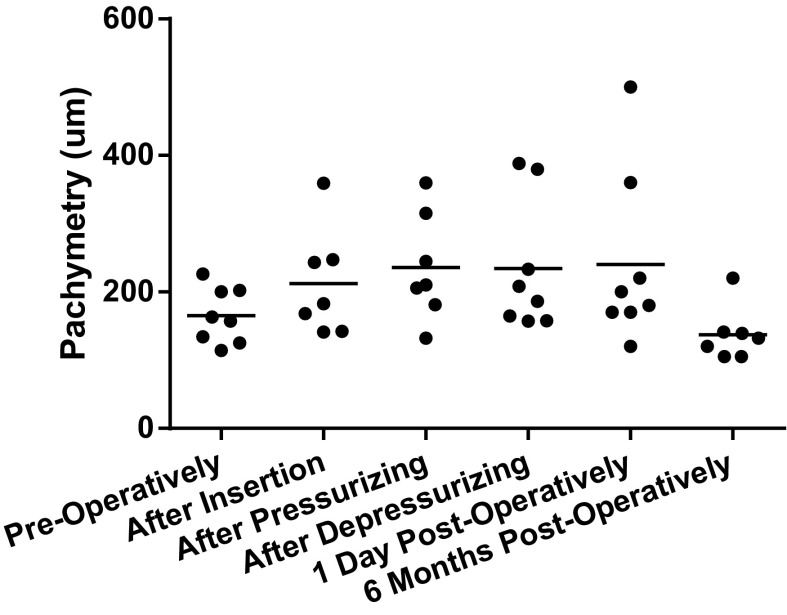
A scatterplot of central graft thickness (in µm) with trend line. Graft thickness was measured before storage by the donor bank, directly after insertion of the graft, directly after pressurizing the eye, after 12 min of pressure when the IOP is normalized, on day 1 post-operatively, and 6 months post-operatively

OCT scans were processed and analyzed using ImageJ, a public-domain Java-based image processing program, developed by the National Institutes of Health [15], recommended by the Zeiss technicians. Statistical analysis of results was performed using SPSS, version 21.0 (IBM, New York, USA) and GraphPad Prism 6 (GraphPad Inc., San Diego, USA). GraphPad Prism 6 was used to produce Graphs 1 and 2. A Shapiro–Wilk analysis was performed to determine the normality of spherical and cylindrical refractive errors, IOP, ECD, and BCVA. To minimize observer bias, graft thickness and interface width were measured by two independent observers (J.S. and F.H.). The mean of both the measurements was used in the analyses.

## Results

### Baseline characteristics

Eight patients (5 males and 3 females) with a mean age of 64.8 years (range 56–74) were included. Seven patients were diagnosed with Fuchs’ endothelial dystrophy and one with pseudophakic bullous keratopathy. All the patients were pseudophakic. The corneal grafts used in this study had a pre-operative mean central thickness of 178 µm (range 114–270) and a mean ECD of 2688 cells/mm^2^ ± 183 (range 2400–3000). Patient and donor characteristics are shown in Table 1.

**Table 1:** Patient and matched donor characteristics

Subject no.	Gender	Age at procedure	Procedure	Eye	Indication	Donor age	ECD	Central graft thickness (µm)
1	Male	60	DSEK	OS	FED	74	3000	134
2	Male	60	DSEK	OS	FED	57	2800	125
3	Female	56	DSEK	OD	FED	39	2800	226
4	Female	69	DSEK	OS	FED	68	2600	200
5	Male	72	DSEK	OD	FED	70	2400	157
6	Male	74	DSEK	OS	FED	62	2700	163
7	Female	63	DSEK	OS	PBK	61	2500	114
8	Male	64	DSEK	OD	FED	72	2600	202

*FED* Fuchs endothelial dystrophy, *PBK* pseudophakic bullous keratopathy, *ECD* endothelial cell density

### Clinical results

Seven surgeries went uneventful. In one case, the lamella inadvertently adhered to the punch, fell off, and the orientation was then impossible to determine, for the stromal side was not marked. In this study, iOCT imaging was inconclusive, and another donor lamella was used. One patient (no. 5) showed a completely dislocated lamella 3 days after surgery. A successful rebubbling procedure was performed. No other post-operative complications (cystoid macula edema, secondary glaucoma) were reported. All the grafts were functional and fully adhered, but one case of bullous pseudophakic keratopathy showed persisting stromal scarring.

Mean Snellen BCVA after 6 months was 0.67 (=logMAR 0.174) (range 0.35–1.00, SD ± 0,25). Mean ECD after 6 months was 1547 ± 573.7 (range 934–2341). Mean increases in the spherical and cylindrical aberrations were −0.25 ± 1.1 (range −1.75–1.25) and −0.56 ± 1.8 (range −4.00–1.25), respectively.

### Study results

Mean pre-operative donor lenticule thickness was 165 µm (range 114–226). At the end of the surgery, i.e., after normalizing the IOP, mean graft thickness was 235 µm (range 157–388), an increase of 42 % compared to pre-operative graft thickness. On day 1 of follow-up, mean central graft thickness was 240 µm (range 120–500), which further decreased to 137 µm ±39.31 (range 105–220) at 6 months post-surgery. The mean interface width between host and graft cornea declined as surgery progressed, and the detailed measurements are reported in Table 2 and Graph 1. In six patients, the iOCT showed some persisting fluid in the interface (no. 2, 3, 5, 6, 7, and 8) after 12 min of pressure, of whom one showed a full lamellar detachment (no. 5). The other cases were successfully managed with corneal swiping with a subsequent decrease of maximal interface width. The interface widths reported are measured after corneal swiping. Prior to swiping, iOCT imaging was performed but no measurements were taken. One day after surgery, all fluid had vanished from the interface. As shown in Graph 2, graft thickness remained stable during surgery and follow-up after an initial increase compared to donor bank-measured thickness.

**Table 2:** Overview of the development of maximum interface width during subsequent stages of DSEK surgery

Subject no.	After insertion (μm)	Directly after pressurizing (μm)	After 12 min of pressure (μm)	1 day post-op (μm)	6 Months post-op (μm)
1	36	42	0	0	0
2	32	23	61	0	0
3	64	54	43	0	0
4	u	u	0	0	0
5	123	84	113	115	0
6	59	34	17	0	0
7	u	26	15	0	0
8	24	26	6	0	u
Mean	56	41	32	14 (median: 0)	0
SD	36	22	39	41	0

*SD* standard deviation, *U* unattainable

## Discussion

This case series describes the use of the Rescan 700 intraoperative OCT surgical microscope in assessing graft–host interface in DSAEK and indicates a potential relationship between interface width observable with iOCT and posterior lamellar detachment. iOCT has the potential to aid the clinician in further lowering the rates of graft dislocation after DSAEK.

We found three distinct advantages of using iOCT in DSAEK surgery: First of all, surgery no longer had to be paused to obtain images, and image stabilization is facilitated by the integration into the microscope, in contrast to the use of hand-held and microscope-mounted iOCT as described by Knecht et al. [5] and Ehlers et al. [6]. Second, iOCT delivers real-time micrometer-resolution imaging in a cross-sectional plane, previously not available to the surgeon. In our experience, the additional visualization obtained by iOCT led to the improvement in surgical decision-making and identification of persisting interfaces, undetectable by the surgical microscope without iOCT. These persisting interfaces could have led to a higher rate of detachment and dislocation if left untreated [16]. Even when visualization of the anterior chamber was affected due to the increased corneal edema whilst pressurizing the eye, iOCT still delivered excellent images of the cornea and graft. The interferometry used for OCT imaging is less affected by scattering of corneal edema [17]. Lastly, the RESCAN 700 is completely safe for intraoperative use since there is no radiation or risk of surgical field contamination.

Some considerations deserve attention. The RESCAN 700 is a surgical microscope technique in development. The surgeon operating in this study complained of eye fatigue when operating with OCT imaging projected into the oculars of the RESCAN 700 for a full day. No software specifically designed by the microscope manufacturer to measure the obtained scans was available at the moment of writing, leading to the use of public-domain calculation tools. These tools do not take into account the type of scan, light scattering, and image magnification, with possible subsequent lesser precision and accuracy of measurements. Furthermore, these tools were not automated, making the results presented here difficult to repeat in clinical practice. In two patients, some intraoperative corneal thickness and interface width measurements were unattainable as noted above. Larger studies are needed to evaluate the correlation between interface width, corneal thickness, and graft detachment with more definitive results and to assess if complication rates decrease when iOCT is used. Different devices were employed to measure lenticule thickness, although the regular anterior segment OCT devices are reported to show excellent repeatability and inter-device agreement [18].

In conclusion, intraoperative OCT is a very promising tool in posterior lamellar keratoplasty. The cross-sectional imaging provides the surgeon with new visual dimensions of the surgical field and potentially improves surgical decision-making. With adequate image analysis tools, iOCT has the potential to be a paradigm-shifting development in anterior segment ocular surgery.
